# Editorial: Women and Entrepreneurship

**DOI:** 10.3389/fpsyg.2021.762028

**Published:** 2021-09-24

**Authors:** Brizeida R. Hernandez-Sánchez, Jose Carlos Sánchez-García, Radha R. Sharma, Antonio Carrizo Moreira

**Affiliations:** ^1^University of Salamanca, Salamanca, Spain; ^2^New Delhi Institute of Management, New Delhi, India; ^3^University of Aveiro, Aveiro, Portugal

**Keywords:** women, entrepreneurship, Korean women, entrepreneurial exposure, entrepreneurial intention

## Introduction

Entrepreneurship is an important driver of economic development and social progress, however, most research has focused on sectors that are male dominated and embody a male perspective (Marlow and McAdam, 2013; Henry et al., 2016), paying little attention to gender issues (Zampetakis et al., 2016).

Although emerging literature has recognized the significant role of women in the broader entrepreneurial phenomenon and economic development (Sarfaraz et al., 2014), there is still a limited understanding of how a female perspective can contribute to entrepreneurship research. For example, there is a paucity of research examining what inspires women to choose an entrepreneurial career, or how female entrepreneurs respond to innovation processes (Brush et al., 2019; Ladge et al., 2019).

In a broader view, the research needs to broaden existing theoretical concepts to better explain the uniqueness that distinguishes female entrepreneurship as an object of independent investigation. This need, however, appears important given the emerging flow of research documenting how men and women choose divergent paths when they decide to pursue a professional career (Ding and Choi, 2011), but also when they find themselves having to play a managerial role (Diaz-Garcia et al., 2013; Kim and Starks, 2016; Ruiz-Jiménez et al., 2016).

For these reasons, this Research Topic has focused attention on the intersection of entrepreneurship and gender, aiming to stimulate academic conversations to build a better understanding of the phenomenon, as well as the challenges that are unique to women and the changes that can be implemented to overturn the phenomenon balance and increase the number of female entrepreneurs.

## Overview of Papers in the Research Topic

The papers included in this Research Topic have adopted a wide variety of perspectives and methodologies. In this sense, the first article of the Research Topic by Cardella et al., have critiqued the extant literature related to gender, highlighting the factors that support women's career choices, and also the prejudices and stereotypes that create barriers to the progress of female entrepreneurship. Through a series of recommendations to educational institutions and research to help women overcome these prejudices, the article aims to highlight the efforts made by researchers for a clearer understanding of the phenomenon, as well as the related challenges that have not yet found an answer.

The second article by Kim analyzed the career motivations that drive Korean women to continue their work despite challenges and the difficulties they face every day; among these, the motivation for career persistence was considered as the most important variable. This study is the maiden attempt to conceptualize the career motivations of married women, and serves as a starting point for future research and practice to improve social perceptions and design support systems for women experiencing work-family to conflict.

The last two articles of this topic focused on the facilitators for entrepreneurial in bridging the gap between men and women. Specifically, the study by Botha has stressed on the importance of previous exposure to entrepreneurial action and on the effect of moderation of some variables namely, leadership, innovativeness, curiosity, self-efficacy and motivation. The results evidenced that previous entrepreneurial exposure is particularly important in encouraging women to start a business, underlining the importance of role models in the transition between intention and action and laying the groundwork for important theoretical and practical implications. The fourth article by Ward et al. analyzed the relationship between entrepreneurial potential, gender and entrepreneurial intention. The results showed that the differences between males and females are not significant, and derive only from intentions, perceived behavioral control (PBC) and subjective norm, which are greater in males, while entrepreneurial motivations are greater in females. Furthermore, the present study underlined the motivational variability that distinguishes men and women in their entrepreneurship orientation. There is important paradigm shift in entrepreneurial research: in both males and females, part of their determination toward business is caused by the need to ensure a workplace, suggesting that it is not always true that entrepreneurship is something that men want and that women choose out of necessity.

## Conclusions

This Research Topic is a collection of articles on female entrepreneurship, however, in particular it addresses the topical issue of the gender gap in entrepreneurship. This unique collection opens up scope for more in-depth research addressing questions such as: How to reduce or overcome the gender gap? What are the factors that support the choice of an entrepreneurial career in women? What are the possible obstacles? How can institutions provide organizational support to foster the participation of women in taking up entrepreneurship. These are only few questions addressed with the current Research Topic and thanks to the variety of perspectives shared by the contributors. We have set the ball rolling and our endeavour is to draw attention to this pivotal and under researched field of women and entrepreneurship by future researchers.

## Author Contributions

All authors have contributed equally to the editing of this manuscript.

## Conflict of Interest

The authors declare that the research was conducted in the absence of any commercial or financial relationships that could be construed as a potential conflict of interest.

## Publisher's Note

All claims expressed in this article are solely those of the authors and do not necessarily represent those of their affiliated organizations, or those of the publisher, the editors and the reviewers. Any product that may be evaluated in this article, or claim that may be made by its manufacturer, is not guaranteed or endorsed by the publisher.

## References

[B1] Brush C. Edelman L. F. Manolova T. Welter F. (2019). A gendered look at entrepreneurship ecosystems. Small Bus. Econ. 53, 393–408. 10.1007/s11187-018-9992-9

[B2] Diaz-Garcia C. GonzalezMoreno A. Saez-Martinez F. J. (2013). Gender diversity within RandD teams: its impact on radicalness of innovation. Innovation 15, 149–160. 10.5172/impp.2013.15.2.149

[B3] Ding W. Choi E. (2011). Divergent paths to commercial science: a comparison of scientists' founding and advising activities. Res. Policy 40, 69–80. 10.1016/j.respol.2010.09.011

[B4] Henry C. Foss L. Ahl H. (2016). Gender and entrepreneurship research: a review of methodological approaches. Int. Small Bus. J. 34, 217–241. 10.1177/0266242614549779

[B5] Kim D. Starks L. T. (2016). Gender diversity on corporate boards: do women contribute unique skills? Am. Econ. Rev. 106, 267–271. 10.1257/aer.p20161032

[B6] Ladge J. Eddleston K. A. Sugiyama K. (2019). Am I an entrepreneur? How imposter fears hinder women entrepreneurs' business growth. Bus. Horiz. 62:5. 10.1016/j.bushor.2019.05.001

[B7] Marlow S. McAdam M. (2013). Gender and entrepreneurship: advancing debate and challenging myths; exploring the mystery of the under-performing female entrepreneur. Int. J. Entrep. Behav. Res. 19:1. 10.1108/13552551311299288

[B8] Ruiz-Jiménez J. M. Fuentes-Fuentes M. Ruiz-Arroyo M. (2016). Knowledge combination capability and innovation: the effects of gender diversity on top management teams in technology-based firms. J. Bus. Ethics 135, 503–515. 10.1007/s10551-014-2462-7

[B9] Sarfaraz L. Faghih N. Majd A. A. (2014). The relationship between women entrepreneurship and gender equality. J. Glob. Entrepr. Res. 4:6. 10.1186/2251-7316-2-6

[B10] Zampetakis L. A. Bakatsaki M. Kafetsios K. Moustakis V. S. (2016). Sex differences in entrepreneurs' business growth intentions: an identity approach. J. Innov. Entrep. 5:29. 10.1186/s13731-016-0057-5

